# COgnitive-Pulmonary Disease

**DOI:** 10.1155/2014/697825

**Published:** 2014-03-16

**Authors:** Fiona A. H. M. Cleutjens, Daisy J. A. Janssen, Rudolf W. H. M. Ponds, Jeanette B. Dijkstra, Emiel F. M. Wouters

**Affiliations:** ^1^Program Development Centre, CIRO+, Centre of Expertise for Chronic Organ Failure, Hornerheide 1, 6085NM Horn, The Netherlands; ^2^Centre of Expertise for Palliative Care, Maastricht UMC+, P Debyelaan 25, 6202 AZ Maastricht, The Netherlands; ^3^Department of Psychology, Maastricht UMC+/School for Mental Health and Neurosciences (MHeNS), Dr. Translaan 12, 6202AZ Maastricht, The Netherlands; ^4^Department of Respiratory Medicine, Maastricht UMC+, P Debyelaan 25, 6202 AZ, Maastricht, The Netherlands

## Abstract

Over the past few decades, chronic obstructive lung disease (COPD) has been considered a disease of the lungs, often caused by smoking. Nowadays, COPD is regarded as a systemic disease. Both physical effects and effects on brains, including impaired psychological and cognitive functioning, have been demonstrated. Patients with COPD may have cognitive impairment, either globally or in single cognitive domains, such as information processing, attention and concentration, memory, executive functioning, and self-control. Possible causes are hypoxemia, hypercapnia, exacerbations, and decreased physical activity. Cognitive impairment in these patients may be related to structural brain abnormalities, such as gray-matter pathologic changes and the loss of white matter integrity which can be induced by smoking. Cognitive impairment can have a negative impact on health and daily life and may be associated with widespread consequences for disease management programs. It is important to assess cognitive functioning in patients with COPD in order to optimize patient-oriented treatment and to reduce personal discomfort, hospital admissions, and mortality. This paper will summarize the current knowledge about cognitive impairment as extrapulmonary feature of COPD. Hereby, the impact of smoking on cognitive functioning and the impact of cognitive impairment on smoking behaviour will be examined.

## 1. Introduction

The twentieth century spread of tobacco use led to a global epidemic of smoking-related illnesses, resulting in 5.4 million deaths worldwide each year. The yearly toll is expected to surpass 6 million by 2015. By then, approximately 30% of the tobacco-related deaths will be caused by chronic respiratory diseases [1]. Cigarette smoking is the leading cause of chronic obstructive pulmonary disease (COPD). The risk of COPD is 56-fold higher in current smokers than in never-smokers [2]. Passive exposure to smoking, air pollution, and occupational chemical fumes or dust may act synergistically with active smoking to increase the risk of COPD [3]. COPD is defined as a disease characterized by progressive, irreversible airflow limitation and abnormal inflammatory response in the lungs [4]. Extrapulmonary features of COPD include weight loss, decrease in muscle mass, strength, and endurance, osteoporosis, heart failure, anxiety, depression, and cognitive impairment [4]. A recent review article indicates a specific pattern of cognitive impairment in patients with COPD [5]. This suggests that COPD is associated with specific abnormalities in brain structure. Cognitive impairment might have an adverse effect on functional, social, emotional, affective, and communication skills. Nowadays, COPD is increasingly seen as a disorder with both physical effects and effects on brains, including impaired psychological and cognitive functioning. The present narrative review provides an overview of the currently available knowledge about cognitive impairment as extrapulmonary feature of COPD. Hereby, the impact of smoking on cognitive functioning and the impact of cognitive impairment on smoking behaviour will be examined.

## 2. Cognitive Functioning

**Figure 1:**
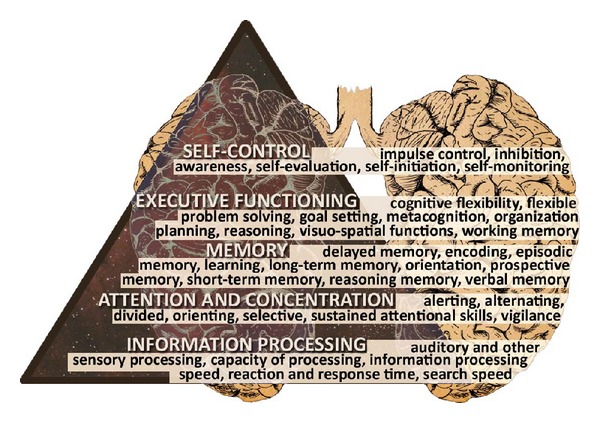
Cognitive domains and specific functions.

Cognitive functioning refers to a range of brain functions that include the processes by which an individual perceives, registers, stores, retrieves, and uses information by which our behaviour can be adapted to new situations. Cognitive functioning can be divided into several cognitive domains, such as information processing, attention and concentration, memory, executive functioning, and self-control. Each domain contains specific functions, which can be seen as basic capabilities that influence the content and amount of intellectual skills, personal knowledge, and competences (Figure 1). These cognitive functions allow us to read, remember a phone number, recognize a human face, drive, and make decisions [6]. The executive functions can be seen as the “higher” cognitive functions such as complex cognitive activities, purposeful, self-regulatory, and future-oriented behaviour (e.g., planning, initiating, and problem solving), involved in the regulation and control of “lower” cognitive functions [7]. Self-control consists of a subset of self-regulatory processes which aim to prevent yielding to unwanted impulses or urges (such as craving for a cigarette when trying to quit smoking) [8].

## 3. COPD = COgnitive-Pulmonary Disease?

In 1992, Grant et al. showed that 42% of the patients with COPD had moderate to severe cognitive impairment compared to only 14% in controls [9]. The incidence of cognitive impairment in patients with COPD varies in different studies from 12 to 88% [10]. Cognitive impairment in patients with severe to very severe COPD seems to be associated with the severity of airway obstruction [11]. However, not all studies show a relationship between FEV_1_ and cognitive functioning [5]. Dodd et al. showed that patients with COPD and an exacerbation had poor scores on cognitive measures compared to stable patients with COPD. This was independent of the presence of hypoxia, vascular risk factors, the number of pack years, and even the severity of COPD [12]. Incalzi et al. showed that the average performance on cognitive tests of patients with COPD was comparable with the performance of patients with vascular dementia [13]. Unfortunately, this paper did not mention the stage of vascular dementia of these subjects. Villeneuve et al. demonstrated that 36% of patients with COPD, compared with 12% of healthy subjects, had mild cognitive impairment, which refers to significant cognitive decline without major functional impacts on activities of daily living. Cognitive impairment may occur in different domains of cognitive functioning [5]. However, the current literature does not provide a clear picture of the pattern of cognitive impairment in COPD. Discrepancies in the current literature can be explained by several methodological limitations of previous studies, such as unknown or self-reported premorbid cognitive functioning [12], limited neuropsychological assessment [14], a self-reported diagnosis of COPD [15], use of control groups that are not matched on potentially important characteristics, for example, educational level [16], or inclusion of patients with COPD without comorbidities [17].

## 4. Causes of Cognitive Impairment in COPD

In recent years the literature on cognitive functioning in COPD has postulated several causes for cognitive impairment, including brain damage, reduced physical activity, and exacerbations.

### 4.1. Atrophy

It is assumed that cognitive impairment in patients with COPD is related to structural brain abnormalities. A voxel-based morphometric study showed that stable patients with COPD in contrast to controls had a lower density of gray matter in the limbic and paralimbic brain regions. This was correlated with a decrease in the arterial oxygen content, deterioration of performance in visual tasks, and an increased duration of illness [18]. Ryu et al. showed that patients with severe COPD showed extensive regions with significantly lower fractional anisotropy (a measure for the quantitative integrity of brain tissue) and higher trace (an index of water movement across cell membrane) in gray and white matter compared to controls. Furthermore, a decrease in fractional anisotropy was demonstrated in prefrontal lobes of persons with moderate COPD compared to normal control subjects [19]. The loss of white matter integrity was associated with the severity of COPD suggesting a correlation with a significant decrease in frontal function in persons with severe COPD and may explain pathophysiological and psychological changes in patients with COPD [18]. Using a Single Photon Emission Computed Tomography (SPECT) scan in patients with COPD without hypoxemia, a decreased perfusion in the left frontal regions of the brain was observed, whereas in patients with COPD and hypoxemia both a decreased perfusion in frontal and parietal areas was found. Reduction of brain perfusion is correlated with impairment in verbal memory, attention, and delayed memory [20]. The brain abnormalities which are seen in patients with COPD are also found in chronic hypoxic diseases such as obstructive sleep apnea syndrome and congenital central hypoventilation syndrome. Possible causes of structural abnormalities in the brains of COPD are cigarette smoke, inflammation, hypoxemia, atherosclerosis, hypercapnia, and nocturnal desaturations.

### 4.2. Smoking

In 2011, the American rapper ASAP Rocky sang “I smoked away my brain,” and in fact, smoke may cause severe brain damage. Smoke from a cigarette, pipe, or cigar consists of many toxic chemicals, including tar, carbon monoxide, and nicotine. Nicotine is believed to be the most active ingredient that modulates brain function and produces addiction [21].

Nicotine inhaled via cigarette smoke enters the bloodstream through the lungs and reaches the brain in 10–20 seconds [22]. Depending on the number, volume, duration, smoke dilution, and depth of inhalation, nicotine can act as either a stimulant or tranquilizer. This can explain why smokers report that smoking gives them energy, stimulates their mental activity, and helps them to attend and concentrate, while others note that smoking relieves anxiety, leads to feelings of contentment, and relaxes them [23]. Upon entering the bloodstream, nicotine immediately stimulates the release of neurotransmitters, neuromodulators, and hormones, which are responsible for most of nicotine's effects like reducing pain, stress, and anxiety or increasing arousal and enhancing cognitive functions like alertness, concentration, and memory [24]. Additionally, there are a lot of adverse neurocognitive effects of cigarette smoking in humans (vide infra).

#### 4.2.1. Acute Nicotine Exposure

**Figure 2:**
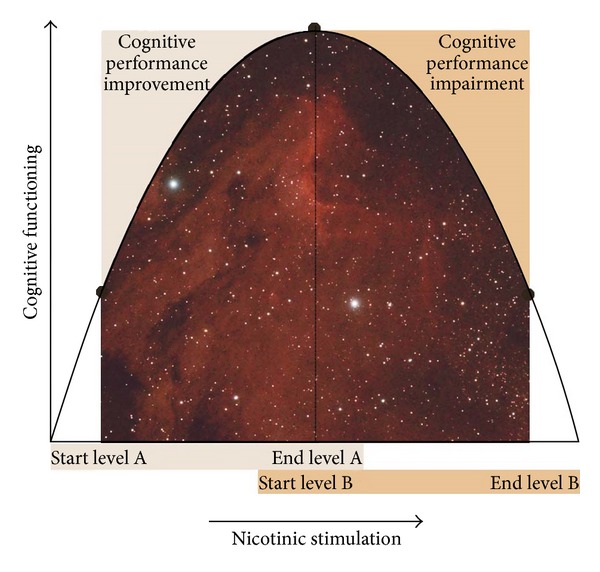
Nicotinic stimulation results are a reflection of baseline cognitive performance level (data from Figure  1 in [25]).

Several studies demonstrated cognitive benefit due to acute nicotine exposure. However, such cognitive enhancement may only be observed in persons with neuropsychiatric disorders (e.g., Alzheimer's disease, attention-deficit hyperactivity disorder, Parkinson's disease, and schizophrenia) who exhibit defined cognitive deficits that are intrinsic to their illness [25]. Improvement in some cognitive domains is mostly seen in vigilance and working memory [26]. Except for the positive gains in alerting attention accuracy and response time, fine motor skills, orienting attention, reaction time, short-term episodic memory accuracy, and working memory reaction time [27], there is no evidence to support nicotinic enhancement of cognitive functioning in healthy adult smokers and nonsmokers. Also under extreme task demands it has been shown that smokers are disadvantaged compared to never-smokers. This suggests that cigarette smoking may work against a person's ability to apply sufficient cognitive resources to achieve maximal performance under progressively more difficult testing conditions [28]. Studies of healthy non- or never-smokers are unlikely to show cognitive performance improvement with nicotinic stimulation by smoking or nicotine patches because they are likely to be operating already at or near optimum levels of cognitive performance. In contrast, studies that tend to show enhancement of cognitive functioning generally utilize persons with impaired cognitive functioning (e.g., neuropsychiatric subjects) or for whom task demands do not match the level of on-going nicotinic stimulation [25]. In these persons nicotinic stimulation brings their cognitive performance to near optimal levels (Figure 2).

#### 4.2.2. Chronic Cigarette Smoking

Chronic cigarette smoking generally does not improve cognitive functioning. Chronicity of smoking is reflected in the number of cigarettes smoked daily, the lifetime duration of smoking, and dose duration (e.g., pack years). A review by Durazzo et al. found poorer domain-specific cognitive skills, including auditory-verbal learning and memory, information processing speed, cognitive flexibility, executive functions, general intelligence, reasoning, sustained attention and impulse control, visual search speeds, and working memory among smokers, compared to nonsmokers [29]. Impairment in specific cognitive domains is found to be positively related to the level of chronicity of smoking [30]. Jacobsen and colleagues observed greater impairment in working memory performance accuracy in adolescents who began smoking at younger age than those who began smoking at older age [31]. These findings implicate a continuum of toxicity, whereby earlier exposure to nicotine is associated with greater brain damage than is exposure occurring at later age. Similarly, in animal models and children, prenatal nicotine exposure causes cognitive impairment [32]. With increasing age, chronic smoking is associated with declined global cognitive functioning [33] and reasoning memory [34] and an increased risk for both vascular dementia and Alzheimer's disease [35]. The risk of dementia is dose dependent and increases with the number of cigarettes smoked [35]. Inconsistencies among studies may be related to the smoking study cohort and the baseline cognitive performance level.

#### 4.2.3. Smoking in COgnitive-Pulmonary Disease

Smoking may promote cerebral atherosclerosis and hypoxemia. It may also affect the microstructural integrity of cerebral white matter due to the stimulating effect of nicotine on nicotine receptors and induction of cerebral small-vessel disease [19]. Consequently, cognitive functioning may be negatively affected. Lung function has been implicated as a potential mediator of the association between smoking and the adverse neurocognitive consequences since research on hypoxemic patients with COPD has documented the presence of mild decrements in cognitive functioning [9]. Because smoking history is a primary risk factor for COPD and other lung disorders, smoking may have an indirect effect on cognitive functioning through its impact on lung function [36]. However, some studies have shown an association between lung function and cognitive function, regardless of smoking status [37].

#### 4.2.4. The Influence of Cognitive Impairment on Smoking Cessation

Common barriers to quitting smoking include concerns regarding weight gain, cost of medicine and classes, discouragement, disruption of relationships, fear of failure, and the loss of perceived psychological benefits of smoking (reward effects, e.g., stress relieve) [38, 39]. After diffusion into brain tissue nicotine binds to nicotinic acetylcholine receptors (nAChRs), for example, the *α*_4_ *β*_2_ receptor subtype [40]. Receptor stimulation results in increased levels of several neurotransmitters, neuromodulators, and hormones in the brain. The neurotransmitter dopamine induces a pleasurable experience and is critical to the reward effects of nicotine [25, 41]. Next to the brain reward effects, craving for cigarettes and withdrawal symptoms (e.g., anxiety, depressed mood, difficulty in concentrating, irritability, restlessness, and sleep/appetite disruption) after quitting serve as a barrier to smoking cessation. Craving may stem from both psychological and physical dependence on nicotine and the associated withdrawal effects can become conditioned cues for smoking. However, conditioning develops only by pairing the pharmacologic actions of nicotine with behaviors. In individuals who are vulnerable to addiction, repeated exposure to nicotine induces neuroadaptation (tolerance) to some of the effects of nicotine. The number of binding sites on the nACHRs in the brain increases, probably in response to nicotine-mediated desensitization of receptors. These neuroadaptive changes are the bases of nicotine tolerance and addiction. Withdrawal symptoms occur in chronic smokers when desensitized *α*_4_ *β*_2_ nAChRs become activated in the absence of nicotine [40]. Taken together, individuals become addicted not only to smoking cigarettes but also to neurotransmitter, neuromodulator, and hormone release in the brain. The desire to quit smoking is not sufficient to accomplish smoking cessation. The ability to guide our behavior according to our long-term goals requires inhibition and monitoring of ongoing behavior [42]. In order to quit smoking, actions should be planned and the determination to implement these actions is crucial. The cognitive domains involved are executive functioning and self-control. These are essential for making a deliberate effort to break habitual and rigid behavioral patterns in order to fulfill the desire to quit smoking. Impairment in executive functioning as seen in older adults has a negative effect on quitting smoking [43].

#### 4.2.5. Genetic Predisposition for Smoking Dependency

The finding that genetic factors account for roughly 70% of the variance in nicotine dependency in adults supports a type of biological vulnerability [44]. Evidence from affected relative or allele sharing methods of analysis demonstrates a number of plausible “candidate genes” for nicotine dependency, which may affect individual's vulnerability to develop nicotine dependency. These candidate genes code for dopamine receptors and transporters, GABA receptors, nAChR-receptor subtypes, and opiate and cannabinoid receptors [45]. In addition, polymorphisms in the CYP2A6 gene are associated with slower nicotine metabolism which reduces the number of cigarettes smoked per day [44]. Furthermore, genes in the chromosomal region that code for brain-derived neurotropic factor (BDNF) are associated with smoking initiation [46]. Genetically based personality traits like impulsivity and risk taking may predispose certain individuals to experiment with drugs, including tobacco [47]. In addition adoption, family, and twin studies converge on the relevance of genetic factors in the use of tobacco and the risk of developing nicotine dependence. Parameter estimates for heritability, shared environmental influences, and unique environmental influences for nicotine dependence are 67%, 2%, and 31%, respectively [48]. Greater concordance in current smoking, experimentation, and cessation has been observed in identical twin pairs compared to fraternal twin pairs [49]. Niu et al. demonstrated that individuals with a nicotine dependent sibling are more likely to be nicotine dependent as well [50]. Adoption studies tried to discriminate between genetic and environmental influences. These studies demonstrated a greater similarity of smoking between adoptive children and their biological parents [51] which suggests a genetic determination of smoking. Although studies confirm the important role of both genetic and environmental influences in nicotine dependence, there is little consistency between studies for the importance of family environment. It is also possible that normative pressure encourages smoking behavior though this might also depend on their particular genetic makeup [52].

### 4.3. Inflammation

Many inflammatory cells, mediators, and enzymes are involved in the complex pathophysiology of COPD. White blood cell count and levels of C-reactive protein, interleukin (IL)-6, and fibrinogen are elevated and tend to increase with disease progression [53]. Inflammation from the systemic effects of COPD may also damage the white matter integrity [12]. C-reactive protein has a direct neurotoxic effect and contributes to cerebral atherosclerosis. Further, IL-6, IL-1*β*, tumor necrosis factor-*α*, and *α*1-antichymotrypsin have been associated with cognitive impairment [54, 55]. The effects of acute, chronic, and systemic inflammation have been associated with cognitive impairment in patients with COPD [12, 37, 56, 57].

### 4.4. Alveolar Hypoxia and Consequent Hypoxemia

Hypoxia is characterized by deprivation of oxygen supply to an organ or the total body. Hypoxic episodes or chronic hypoxia in the brain can lead to the generation of free radicals, an inflammatory mediated neurotoxic effect, and oxygen dependent enzyme, which mediate the neuronal damage. [19]. Although some studies found weak or no correlation between cognitive functioning and hypoxia, there are studies that show that patients with COPD and hypoxia have more cognitive impairments than patients with COPD without hypoxia [9, 11]. Likewise, hypoxemia, which is characterized by low levels of dissolved oxygen in the arterial blood, can result in a lack of oxygen supply to the brains and may lead to cognitive impairment in attention, reasoning, memory, and processing speed in patients with COPD [20]. Furthermore, nocturnal desaturations may affect cognitive functioning. It is assumed that the structural abnormalities in the cerebral cortex and white matter result from cerebral hypoperfusion and microemboli, resulting in hypoxia [58]. Thirty years ago it was demonstrated that oxygen supplementation in COPD patients with hypoxemia may have beneficial effects on cognitive functioning [14, 59].

### 4.5. Hypercapnia

As hypoxia, hypercapnia (characterized by increased carbon dioxide levels in the blood) can also lead to the generation of free radicals and oxygen dependent enzymes which can result in global neuronal injury. However, little is known about the relationship between hypercapnia and cognitive impairment in patients with COPD. Although a correlation has been demonstrated between increased arterial carbon dioxide tension and impaired cognitive functioning in attention and memory [60], not all studies observe a correlation between hypercapnia and cognitive functioning [61].

### 4.6. Atherosclerosis

Atherosclerosis is a progressive disease process involving both chronic artery wall inflammation and hypoxia. Atherosclerosis can lead to partial or complete obstruction of the vasculature in the brain. This may lead to oxygen deprivation of brain cells situated behind the obstructed vessel wall, resulting in decreased brain function or even brain cell death (stroke) which can impair cognitive functioning. In patients with cardiovascular disease, the thickness of the carotid artery—a measure of atherosclerosis—is associated with reduced results on cognitive tasks related to attention and executive functions [62]. COPD is known to facilitate atherosclerosis in blood vessels throughout the body, for example, via oxidative stress, hypoxia, hypoxemia, and systemic inflammation [63].

### 4.7. Decreased Physical Activity

Age- and disease-related decline in physical activity is associated with cognitive functioning [64]. Physical activity has a positive effect on cognitive functioning in patients with COPD by influencing mediating factors such as anxiety, depression, nutrition, and sleep quality [65]. Improvement of cognitive functioning after physical activity has been found using a cognitive test of verbal fluency [66]. A vicious circle of deconditioning may occur, whereby cognitive impairment leads to a decrease in physical activity because patients have difficulty with initiating activities or fail to recognize the importance of exercise. Consequently, this could lead to more cognitive deficits.

### 4.8. Exacerbations

During exacerbations, hypoxemia and systemic inflammation increase which may explain cognitive impairment in COPD. Dodd et al. demonstrated worse performance on cognitive functioning tests concerning episodic memory, executive functions, visuospatial functions, working memory, and processing speed in COPD patients with an exacerbation compared to age-matched controls. They also had worse scores compared to stable patients with COPD, except performance on episodic memory and executive function tests. Furthermore, they had lower scores compared to the normal range of the healthy population [12]. While one study showed that cognitive functioning did not improve three months after the exacerbation, other studies showed that cognitive functioning is reversible after six months [12, 67, 68].

### 4.9. Discrepancies and Ambiguities

Although evidence has been found for the above described causes of cognitive impairment in patients with COPD, these factors cannot explain all cognitive impairments [37]. To date, it is still unknown which other factors play a role. Previous research has shown that cerebrovascular disease explains part of the variance in cognitive impairments in patients with COPD and an exacerbation [12]. The variance in cognitive impairment in COPD could also be explained by comorbid conditions such as obstructive sleep apnoea syndrome and major depressive disorders which also decrease cognitive performance [69, 70]. Therefore, causes of cognitive impairment in patients with COPD may be multifactorial.

## 5. Consequences of Cognitive Impairment in COPD

The extent to which a person experiences difficulties in daily life due to cognitive impairment depends on which cognitive functions are affected. In theory, “higher” cognitive functions affect the operation of “lower” cognitive functions. Three consequences of cognitive impairment can be distinguished. First, immediate discomfort for the patient, such as memory problems and problems with attention and concentration, occurs [37]. Second, cognitive impairment affects self-management. Third, cognitive functioning affects the duration and frequency of hospital admissions and mortality [71].

Patients with COPD will be advised to stop smoking, use their medication properly, and maintain an active lifestyle [72]. Furthermore, cognitive impairment is related to limitations in activities of daily living. A study of Antonelli Incalzi et al. demonstrated an association in patients with COPD between low results on cognitive tests and disability in activities of daily life [73]. Physical activity patterns of patients with COPD are usually lower than the ones that the patient is expected to be capable of [74]. This may be explained by the fact that patients can have a cognitive impairment, which limits the ability to initiate activities and understand the importance of activities such as physical exercise. Executive function deficits in patients with COPD lead to improper use of (inhalation) medication, difficulties in dealing with comorbidities, and difficulty in handling guidelines. In addition, patients with executive function deficits seem unmotivated because they do not follow advised guidelines, while they may not have good self-management skills [75]. Consequently, cognitive impairment in patients with COPD may adversely affect treatment. Reduced verbal memory may reduce medication compliance. Poor adherence to medication increases the risk of an acute exacerbation, which consequently results in poorer health outcomes [12].

### 5.1. Hospitalizations and Deaths

A prospective study showed over a three-year period that the coexistence of COPD and cognitive impairment has an additive effect on respiratory-related hospital admissions and mortality [71]. A retrospective study showed that patients who were still alive after three years had a better performance on cognitive tests at the start of the study [76]. The cause still remains unknown. A possible explanation is that cognitive impairment may be more common in patients with severe COPD, hypoxia, inflammation, or comorbidities. Another explanation is that patients with cognitive impairment are more often hospitalized due to insufficient self-management compared to COPD patients without cognitive impairment. The length of hospitalization is also correlated with cognitive functioning, such as processing speed, immediate visual recognition, and verbal fluency [12]. However, cognitive functioning is not a part of the current prognostic indices, such as the BODE index [77].

## 6. Clinical Implications of Cognitive Impairment in Patients with COPD and Conclusions

Cognitive impairment in patients with COPD may affect health and daily functioning. Further research into the degree of cognitive impairment, the origin, and consequences is important to optimize clinical care, treatment, and support of patients with COPD and to reduce exacerbations and hospitalizations, to optimize survival and improve quality of life. In addition, knowledge of deficits in executive functions may lead to insight into continued smoking among persons with respiratory diseases. It also allows clinicians to arrange resources like assistance from a social network and environmental structure in order to enhance the success in quitting smoking. Research on changes in brain morphology, clinical and demographic characteristics of patients with COPD, and comorbid cognitive impairment may give more insight into the risk factors and causes of domain-specific cognitive impairment. By adapting the environment to the personal needs of the patient limitations in daily life can be reduced. Also, education of beneficial health activities and treatment adherence can be provided and the patient can learn to make their own decisions. To maintain the motivation of the patient, it is important to encourage the confidence of the patient and to establish or maintain considerable external support. Hereby it is important to monitor the emotional state of the patient and to have good communication with health care providers, partners, and neighbors. By properly using this system, social support, knowledge of the disease and care, coping, and self-management skills can contribute to improving the care and guidance of these patients. Self-management can be focused on the individual needs and problems in daily practice, knowledge about lung disease, and physical activity. Future studies should focus on interventions with the aim to optimize cognitive functioning of patients with COPD (COgnitive-Pulmonary Disease).
